# Insights into the Angoff method: results from a simulation study

**DOI:** 10.1186/s12909-016-0656-7

**Published:** 2016-05-04

**Authors:** Boaz Shulruf, Tim Wilkinson, Jennifer Weller, Philip Jones, Phillippa Poole

**Affiliations:** University of New South Wales, Sydney, Australia; University of Auckland, Auckland, New Zealand; Otago University Christchurch, Christchurch, New Zealand

## Abstract

**Background:**

In standard setting techniques involving panels of judges, the attributes of judges may affect the cut-scores. This simulation study modelled the effect of the number of judges and test items, as well as the impact of judges’ *attributes* such as accuracy, stringency and influence on others on the *precision* of the cut-scores.

**Methods:**

Forty nine combinations of Angoff panels (*N* = 5, 10, 15, 20, 30, 50, and 80) and test items (*n* = 5, 10, 15, 20, 30, 50, and 80) were simulated. Each combination was simulated 100 times (in total 4,900 simulations). The simulation was of judges attributes: stringency, accuracy and leadership. Impact of judges attributes, number of judges, number of test items and Angoff’s second (compared to the first) round on the precision of a panel’s cut-score was measured by the deviation of the panel’s cut-score from the cut-score’s true value.

**Results:**

Findings from 4900 simulated panels supported Angoff being both reliable and valid. Unless the number of test items is small, panels of around 15 judges with mixed levels of expertise provide the most precise estimates. Furthermore, if test data were not presented, a second round of decision-making, as used in the modified Angoff, adds little to precision. A panel which has only experts or only non-experts yields a cut-score which is less precise than a cut-score yielded by a mixed-expertise panel, suggesting that optimal composition of an Angoff panel should include a range of judges with diverse expertise and stringency.

**Conclusions:**

Simulations aim to improve our understanding of the models assessed but they do not describe natural phenomena as they do not use observed data. While the simulations undertaken in this study help clarify how to set cut-scores defensibly, it is essential to confirm these theories in practice.

## Background

Standard setting is an important aspect of assessment, with the literature describing a plethora of methods. Although each has unique features, most standard setting methods use panels of expert judges to determine the cut-scores between the different performance categories [4, 5, 14, 47, 50]. Among the judge-based standard setting methods, Angoff’s method (henceforth Angoff) and variants, have been used in a range of educational settings [2, 4, 13, 59]. Commonly, Angoff is a process used to estimate performance standards at the pass-fail level; i.e. a process aiming to ‘separate the competent from the non-competent candidate’ ([5], p. 120). In this process, each judge estimates the proportion of minimally competent examinees who would give a correct answer to each of the items. Those estimates are then summed across items for each judge, with the average of the sums across judges determining the test cut-score [2]. A variant, the modified Angoff, includes a second round of judgements after the judges have seen their peers’ judgements. This has been shown to increase inter-judge agreement [13]. Furthermore, [15] demonstrated that Angoff group discussion, which did not include test results, decreased the variance of within-panel estimation of the proportion of correct responses per item; however these discussions did not decrease the differences between the judges’ estimates and the observed proportion of correct values.

Research on the utility of Angoff suggests that the cut-scores generated by a panel are affected by the panel’s composition, particularly the number of judges and their levels of expertise [7, 17, 32, 66, 70]. Numerous modifications have been introduced to the original Angoff method in order to improve the defensibility of the resulting cut-scores [7, 21, 37, 38, 54, 55]. These modifications include providing additional information to the judges, such as judgements made by peers, normative examinee data or pre-judgement training [13, 54]. However, there remains uncertainty about the relative impacts of the number of test items and judges, or judges’ attributes, on cut-scores. The recommended number of judges for Angoff ranges from 5 to 30 [17, 26, 32, 33, 44, 48]. Nonetheless, [36] demonstrated that if judges were randomly sampled from a large pool of qualified judges, at least 87 judges were needed, with 95% probability, to ensure that the cut-score estimation error did not exceed one test item. The impact of the number of test items on the cut-score appeared to be small [26, 33] even when subsets of items taken from the same tests were considered [25]. The impact of the number of items on the validity of the cut-score determined by Angoff panels has not been widely studied. A common view is that the resulting cut-score will be more accurate as the subject expertise of the judges increases; nonetheless, that assertion has not been confirmed empirically [10, 36, 64, 66, 70].

A major challenge in the literature on standard setting is that there is no ‘gold standard’ for standard setting [71]. Furthermore, the Consensus Statement and recommendations from the Ottawa 2010 Conference suggest that validating an assessment by comparing one assessment criterion with another has ‘lost ground,’ since the other assessment criterion also needs validation. This is, in effect, an endless or perpetual process [56]. With the exception of a rough estimate of expertise, i.e. experts vs. non-experts, most evidence on the quality of standard setting is extracted from data from different tests and using different panels where judges’ attributes were not measured.

The current study aims to explore the potential effect of panel constitution and expertise on standard setting. In particular, this study models the impact of the number of judges, the number of items and judges’ *attributes* on the *precision* of the resulting cut-scores as well as the impact of a second round of Angoff on the precision of the cut-score. The research questions are:

1. Is there an optimal number of judges and items for the Angoff standard setting process?
2. What is the impact of a judge’s *attributes* on the *precision* of the cut-score?
3. To what extent does the second round of decision-making (where judges’ decisions are affected by the composition and decisions of other panellists, but not by test parameters) improve the *precision* of the cut-score?

## Methods

To address these questions, this study used simulated data. By their nature, simulated data, a priori, establish the correct (true) value for the cut-score, and hence provide accurate and valid criterion validity [56] for assessing a model [22] of a standard setting method, in this case the Angoff method. For clarity, two cut-scores are discussed in this manuscript: (1) the ‘true’ cut score which is determined by the simulation as described below; and (2) the ‘cut-score’ which is yielded from the simulated judges’ decisions.

By using simulated data, it is possible to compare the cut-scores determined by Angoff panels of simulated judges with the ‘true’ cut-score as set by the simulation parameters. Having a ‘true’ cut-score means that two fundamental assumptions must underlie this study: (1) there is a cut-score that distinguishes competence from incompetence [5], for example the common definition ‘minimally competent examinee’ ([73], p. 219); (2) an examinee must be either competent or incompetent, but cannot be both or neither.

This study simulates judges’ attributes and the impact of these attributes on the cut-score yielded from the Angoff method. To simulate the effect of *attributes* on the *precision* of the cut-score required the generation of a judge’s cut-score for each item. This was made under some assumptions:

- A judge’s expertise was assumed to be positively associated with greater accuracy (i.e. the smaller the deviation of the judge’s cut-score from the true cut-score, the more expert the judges were); thus ‘Accuracy’ in this study is equivalent to expertise;
- Based on previous evidence [64, 66, 70] experts are regarded as more stringent than novices;
- Experts are more likely to have greater influence within the panel [10], which is designated as ‘Leadership’;
- A judge’s estimation of the cut-score is affected by a combination of their personal attributes and a random error [53]. Note that random errors are independent, normally distributed around the true value, with their sum equal or very close to zero [31];
- A judge’s *attributes* are independent of item difficulty [24, 27, 31, 52, 60]. As level of expertise and content-specific knowledge impact on judges’ decisions [66], level of expertise was included in the analysis;
- There is no predefined way of determining the relative impact of stringency and accuracy on a judge’s decisions. This assumption was made since no evidence was found in the literature suggesting otherwise;
- ‘Leadership’ (influence of one judge on others in the second round) is associated with two independent components: the first is a general social attribute of leadership which is independent of expertise; the second is related to expertise, since judges are likely to change their views based on information deemed to be correct, as do experts [10, 16].

The *attributes* that were simulated in this study included:

1. Accuracy: the accuracy of a judge’s cut-score was simulated by varying the size of the random error component in the judge’s cut-score [23].
2. Stringency: the extent to which a judge’s cut-score is affected by bias by being more stringent or lenient was simulated by adding (subtracting) a systematic error to all judges’ decisions where the size of the error determines the level of stringency/leniency [23].
3. Leadership: The extent to which a judge’s cut-score in the second Angoff round is influenced by the cut-scores determined by other judges was simulated by determining the relative contribution (using weighted average) of each judge to the panel’s overall decision. This attribute could be independent of, or associated with, level of expertise. These associations are discussed further below.
4. The relative impact of Stringency and Accuracy on a judge’s decisions: the extent to which each of these attributes (Stringency and Accuracy) affects or dominates the judge’s decision on the cut-score was simulated by the proportional impact of each attribute. These simulation parameters were independent and were included in the simulation to allow different impacts of Stringency and Accuracy on judges’ decision.

The simulation applied standardised measures whereby the correct (true) cut-score for each item (and consequently for the whole test) was set to zero. Hence this study measured only the judges’ decisions and not the difficulty of the items.

The data simulation and data analysis were undertaken using SPSS V22. Data generation parameters used in this simulation are described in Table 1. This table summarises how a judge’s *attributes* were simulated.

**Table 1:** Simulation parameters for generating a score made by Judge j for item i

Notation	Parameter	Random function
A	Judge’s Accuracy	Normal (μ = 0; σ = 1)
S	Judge’s Stringency	Normal (μ = 0; σ = 1)
L	Judge’s Leadership	Normal (μ = 0; σ = 1)
Wa	Accuracy’s Weight	(Normal (μ = 0; σ = 1) + 3)/3
Ws	Stringency’s Weight	(Normal (μ = 0; σ = 1) + 3)/3
Ji	Judge’s raw score of item i	Normal (μ = 0; σ = A_j_)
Sji	Judge’s Stringency for item i	Normal (μ = S_j_; σ = 1)
Sa	The impacts of Accuracy on Stringency^a^	Normal (μ = 1-A_j_; σ = 1)
La	The impacts of Accuracy on Leadership^a^	Normal (μ = 1-A_j_; σ = 1)
Lji	Judge’s Leadership in the second round	Normal (μ = L_j_; σ = 1)

^a^Impact of accuracy is the component of Accuracy (expertise) that contributes to Stringency or Leadership

Formulae for calculating the judges’ mean score for item_i_, which always has a *true* value = 0, are described below. Score1 is the cut-score generated by the panel in round 1 and Score2 is the cut-score generated by the panel in round 2 when only impact of other judges i.e. ‘Leadership’ was added to Score1. Other possible impacts (e.g. students’ actual results) used in different versions of Modified Angoff [37, 49] were not simulated in this study.

It was necessary to simulate weights for Leadership since the second round differed from the first round only by Leadership. ‘Leadership’ was manifested by the weight given to a particular judge’s decision when averaging the panel’s cut-score.

The *attributes* described above were generated for each judge within each panel and used to generate that judge’s scores for each item. Note that these determinants were constant (although derived from a random number function) across all scores for all test items given by the same judge as they described judge’s *attributes* only, but not the scores given by the judge, which was a random number generated by those determinants.

$$ \mathrm{Score}1=\left[{\displaystyle {\sum}_{{}_{\left(\mathrm{j}=1\ \mathrm{t}\mathrm{o}\ \mathrm{n};\ \mathrm{i}=1\ \mathrm{t}\mathrm{o}\ \mathrm{k}\right)}}}\right.\left[\left({\mathrm{Ji}}_{\mathrm{ji}}*{\mathrm{Wa}}_{\mathrm{ji}}+\upmu \left({\mathrm{S}}_{\mathrm{ji}},\ {\mathrm{S}\mathrm{a}}_{\mathrm{ji}}\right)*{\mathrm{Ws}}_{\mathrm{ji}}\right)/{\displaystyle \sum \left({\mathrm{Wa}}_{\mathrm{ji}},\ {\mathrm{Ws}}_{\mathrm{ji}}\right)}\right]/\left(\mathrm{n}*\mathrm{k}\right) $$

$$ \mathrm{Overall}\ \mathrm{Leadership}\ \mathrm{of}\ {\mathrm{j}\mathrm{udge}}_{\mathrm{j}}=\upmu \left(\left({\mathrm{L}}_{\mathrm{j}\mathrm{i}},\ {\mathrm{L}\mathrm{a}}_{\mathrm{j}\mathrm{i}}\right) + 3\right)/3 $$

$$ \mathrm{Score}2=\left[{\displaystyle {\sum}_{{}_{\left(\mathrm{j}=1\ \mathrm{t}\mathrm{o}\ \mathrm{n};\ \mathrm{i}=1\ \mathrm{t}\mathrm{o}\ \mathrm{k}\right)}}}\left(\mathrm{Score}{1}_{\mathrm{ji}}*\left[\upmu \left(\left({\mathrm{L}}_{\mathrm{ji}},\ {\mathrm{L}\mathrm{a}}_{\mathrm{ji}}\right) + 3\right)/3\right.\right)\right]/\left(\mathrm{n}*\mathrm{k}\right) $$

n = no. of judges; k = no. of items

All simulation parameters were standardised to have standard deviation of 1 and mean of 0 or 1 as appropriately required. The results generated by the simulations present the effect of the judges’ *attributes* on the precision (deviation from the true cut-score) of the resulting cut-scores, which was set to zero in all simulations.

The Angoff simulations were performed for a range of judges (5, 10, 15, 20, 30, 50, and 80) and a range of items (5, 10, 15, 20, 30, 50, and 80). These choices were based on previous research showing that the addition of items or judges has greater impact when the numbers are small and the association is not linear [26, 33]. For each simulation, a new panel was generated where each judge had a unique set of *attributes*.

Overall there were 49 simulation combinations, each comprising 100 simulations.

### Statistical analysis

The means and 95 % CI of the means of the cut-scores derived from the 49 sets of 100 panels were calculated and compared. A paired t-test was used to measure the difference in Angoff cut-score precision between the two rounds. Graphical presentations were used to demonstrate the interactions between judges’ *attributes* and *precision*.

### Ethical statement

This study used simulated data only thus no ethical approval was required.

## Results

The results were derived from 4,900 simulated Angoff panels comprising 100 simulations of each of the 49 combinations of the number of judges and of items.

**Fig 1:**
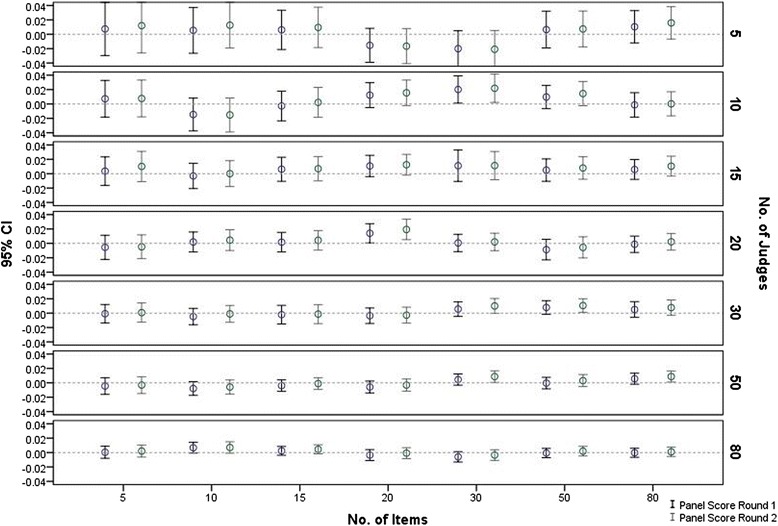
Panels’ mean score (95 % CI) by no. of judges, no. of items & Angoff round
  Figure 2 shows the effect of increasing the number of judges on the *precision* of the panels’ cut-scores for tests with different number of items. For tests with 10 or fewer items, increasing the number of judges significantly improves the *precision*, although there is not more to be gained when the number of judges exceeds 30. For larger tests, increasing the number of judges beyond 15–20 has little effect on improving *precision*.

1. * Is there an optimal number of judges and items for the Angoff standard setting process*?
  The results (Fig. 1) show that the 95 % CI of the mean cut-scores for each of the 100 panels in all judge-item combinations included the true score (zero). There was no relationship observed between the mean cut-score and the number of judges or items. This lack of relationship was evident in the cut-scores both from round one and round two. However, increasing the number of judges was associated with narrower confidence intervals, irrespective of the number of items.

**Fig 2:**
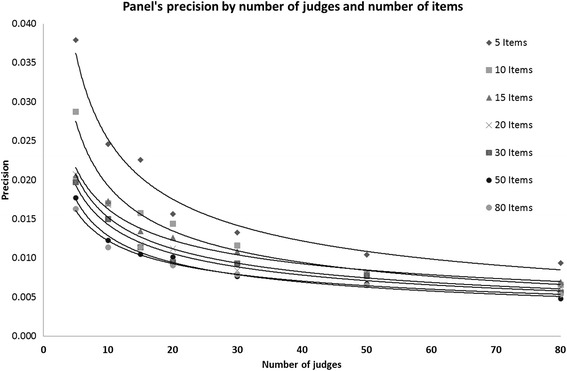
Panels’ precision by no. of judges and no. of items
  Note that in these analyses (Figs. 1 and 2), judges’ *attributes* are not considered and they had no impact since, based on the simulation parameters, their overall impact on any set of 100 panels is equal or very close to zero.
2. * What is the impact of judges’ attributes on the precision of the Angoff cut-score*?
  The first attempt to answer this question was made by measuring the partial correlation (controlled for number of judges and number of items) between the means of the panel’s cut-scores and the means of within-panel judges’ *attributes* (Accuracy, Stringency and Leadership). The correlations were negligible and statistically insignificant. The panels were then classified into deciles (each consisting of 490 panels) based on judges’ *attributes*, to allow identification of any non-linear association. Based on the simulation parameters, the impact of the number of judges and number of items within each decile is very close to zero, thus there was no need to control for those variables.
  The results as shown in Fig. 3 indicate that the association between judges’ *attributes* and the *precision* of the cut-score is complex and non-linear. Figure 3 demonstrates that panels’ cut-scores are most precise (i.e. closer to the true value of zero) when the judges are neither too stringent nor too lenient. However, the interaction of judges’ Accuracy and Stringency showed that when the panel is neither too stringent nor too lenient, the impact of panels’ mean Accuracy (i.e. the within panel agreement) on the cut-score *precision* is small.

**Fig 3:**
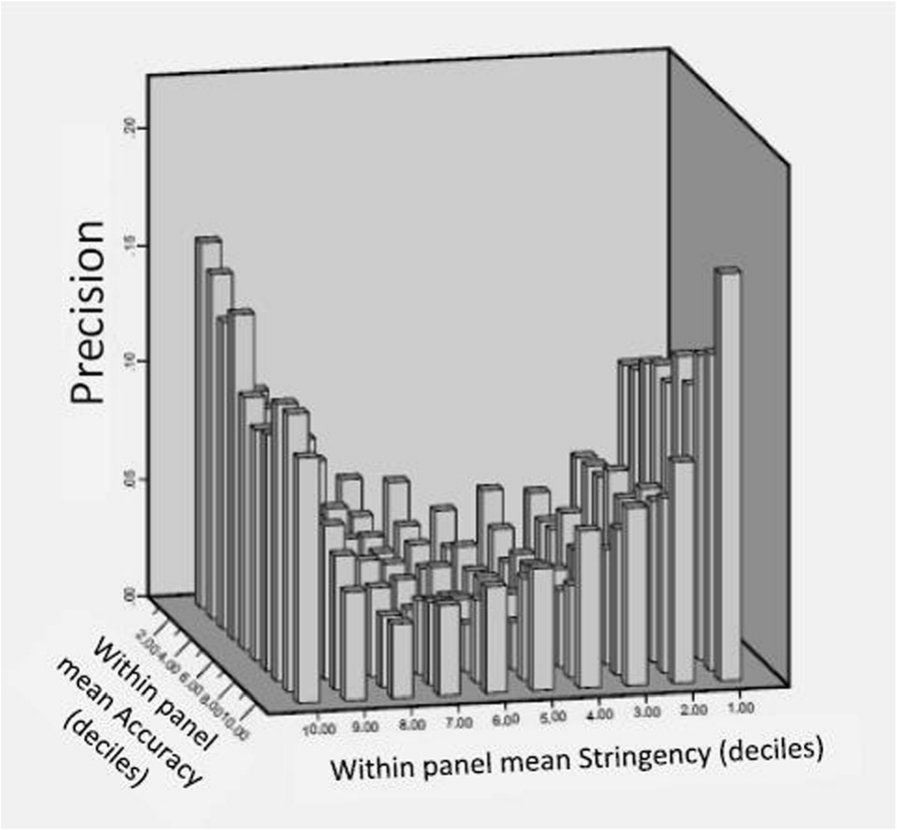
Panels cut-score accuracy by within-panel judges’ Accuracy and Stringency
3. * To what extent does the second round of decision-making improve the precision of the Angoff cut-score*?
  A paired t-test was employed to measure the difference between absolute cut-scores from the two rounds. The difference was statistically significant but practically negligible (Round 1 = .060; Round 2 = .0613; *N* = 4900; *p* < .001; Cohen’s d = −0.083).
  Additional analysis measured the correlation between judges’ level of agreement (SD within panel) and panel precision (cut-score in absolute values). Partial correlation (controlling for number of judges and number of items) between within-panel SD and panel precision (cut-score absolute value) was used to measure the impact of judges’ agreement on the panel cut-score *precision*. The correlation was statistically significant but low (*r* = .226, *p* < .0001) indicating that, although there was a correlation, the within-panel SD explained only 5.1 % of the variance in the cut-score *precision*.

## Discussion

This study used simulated data based on 4900 unique panels of judges, which allowed measurement of the difference between the panels’ determined Angoff cut-scores and the ‘true’ cut-score. The main findings were:

- Increasing the number of judges reduces the variation in the panel’s cut-scores but, more importantly, also increases the *precision* of the panel’s cut-score; however, the effect on *precision* was less evident for tests with a large number of items;
- Judges’ Stringency and, to a lesser effect, judges’ Accuracy affect the cut-score *precision*; and
- Applying the second round of Angoff process without consideration of examinees or test data does not have a meaningful impact on cut-score *precision*.

The findings are discussed in three sections. The first discusses the merit and the appropriateness of the simulation; the second discusses the findings and their implications for researchers and practitioners; and the third section discusses the merit and limitations of this study and possible directions for further research.

### The simulation

Simulated data have been used previously in educational assessment research for knowledge-based tests [18, 34, 52, 60, 62] and for performance-based assessment [40]. However, simulation studies in the field of standard setting are scarce and none was found that simulated judges’ decisions based on their simulated attributes and comparing them with a simulated ‘true value’ [7, 34, 45, 72]. Most previous simulation studies in this field simulated student performance/ examination scores to be used by Angoff panels comprising real judges, yet none of these studies measured judges’ attributes and their impact on the cut-score precision [7, 74]. B. Clauser et al. [15] compared the judges’ estimates of proportion correct answers with empirical data of examinees’ proportion correct answers. This approach, although important, measures the judges’ ability to estimate examinees’ performance on a particular test, but without any empirical evidence to suggest the cut-score that distinguished competence from incompetence [59]. The current study builds upon previous works [3, 15, 33, 43, 61] and extends the use of simulation in this field by simulating judges’ *attributes* that are assumed to affect their decisions, as well as measuring the *precision* of the cut-score by comparing the panels’ determined cut-score with the ‘true’ cut-score.

All previous studies identified in the literature used the variance within judges (or agreement among) as a measure of accuracy or precision. Using such a measure means that if a panel of judges was very stringent but all agreed with each other their agreed cut-score would be deemed more accurate than a cut score yielded by a balanced panel comprising some stringent and some lenient judges, which naturally would yield a larger variance. In real life there is no way to know the true cut-score that distinguishes between competence and incompetence, hence standard setting is employed. For example ([11], p. 158) presented data showing that three different panels estimating the same items yield different agreed cut-scores and different inter-rater variance even when using the same standard setting method (Angoff or Nedelsky). Other studies, (e.g. [17, 33, 37, 48, 65]) which used generalizability analysis to measure the replicability of an Angoff procedure, concluded that a large portion of the overall error variance came from the judges, yet they had no gold standard with which to measure deviation from the true cut-score. This is obvious since generalisability analysis is based on sources of errors while assuming that the mean is very close to the true score [9]. When measuring the precision of a standard setting process, simulation studies like the one presented in this paper, have the unique advantage of including the true cut-score as a valid standard for comparison [58].

The rationale justifying the simulation of each of the variables is discussed in detail in the Method section and not repeated here. However, is it valid to simulate judges’ attributes? Verheggen et al. [64] demonstrated that in standard-setting, a judge’s individual decision on an individual item reflect the ‘inherent stringency of the judge and his/her subject-related knowledge’ ([64], p. 209). This notion was widely mentioned in the literature [17, 66, 70]. Thus, in measurement terms [30], if all items are equally difficult (i.e. difficulty level =0) then the resulting cut-score is comprised of the sum of biases i.e. Judges’ Stringency and sum of random errors i.e. Accuracy and other random errors. Since previous studies suggest that experts are more stringent than non-experts, [64, 66, 70] and are deemed to have greater influence within the panel [10], we included these assumptions in the simulation parameters. The absolute extent to which each of the attributes affects the judgement is unknown, thus the simulation was comprised of standardised parameters (SD ≅ 1) to allow the relative impacts of each parameter on the cut-scores to be ascertained. Note that like all simulation studies, the current study measures interactions for given simulated conditions, for better understanding of an assessment model. This study is not about measuring nature [22]. However, this study is similar to research using real data, in that one study measures impact observed on a particular sample and a different study applies similar measures on a different sample. Often the results are different, yet the difference does not suggest that one study is more correct than the other. Given the concordance with previous studies that used real data [33], it is suggested that the results of this simulation study would be applicable to any population of judges with attributes not unlike what was simulated in this study.

Overall, a simulation study always yields results which are determined by the simulation parameters. The contribution of this study to the standard setting literature is that it measures the impact of judges’ *attributes* at the individual level on the *precision* of the panel’s cut-score. To our knowledge, these associations have never been measured before, either by using simulated or observed data. The concordance of the results of this study with previous studies, particularly where results could be compared (e.g. Fig. 2 vs. work of Hurtz and Hertz [33], Fig. 1 ), support the validity of the simulation assumptions and parameters, thus adding strength to the study findings.

### Implications of the results

Angoff is often used to set standards in large scale educational assessments [8, 59]. Within the context of medical education, Angoff has been applied to tests of medical knowledge (e.g. MCQ’s ) [28, 49], or clinical skills examinations (e.g. OSCE) [20, 37, 55].

In clinical examinations (e.g. OSCE), the number of items (or stations) may be between 10 and 20 [6]. Thus, given that increasing the number of items is unlikely, for reasons of feasibility, our results suggest that if Angoff were used, an optimum combination would be about 30 judges for 10 items, with a minimum of 20 judges for 15 items or more. For MCQs, where the number of items is large [69], a minimum of 15 judges should suffice for setting up a defensible Angoff cut-score for examinations consisting of 80 items or more (Fig. 2). It is noted that increasing the number of items provided more data points , thus higher reliability [1] and therefore also is likely to increase precision.

These findings are within the range recommended in the literature, suggesting that an acceptable cut-score could be reached if 5–25 judges were employed [17, 33, 41, 46, 48]. Since there is no gold standard for any definition of ‘what is good enough’ in standard setting [19, 76], applying Angoff with different numbers of judges might be justifiable depending on the context of the examinations.

Previous studies using observed data have determined Angoff precision by the variance across the judges [37, 48]. Other studies that used observed data used IRT parameters or cut-scores generated by alternative methods to estimate the quality of the Angoff generated cut-scores [63, 75]. These methods are appropriate when observed data are used. In the current study, *precision* was determined by the deviation of the panel’s cut-score from the ’true’ cut-score. The difference between these definitions is more than semantic. Jalili et al. [37] and others [17, 51] used indirect measures to estimate validity as for example, Jalili et al. [37] stated ‘We do not have a reference standard by which to test validity’. Their elegant solution was to use correlation between the panels’ cut-scores and mean observed scores (scores given to examinees by the examiners) for each item as a measure for estimating validity. The current study has the advantage of having a reference standard by which to test validity since it was included in the simulation parameters (true cut-score = 0). Our finding that the correlation was low (*r* = .226, *p* < .0001) indicates that although there was a correlation, the within-panel SD (judges agreement) explained only 5.1 % of the variance in the cut-score *precision*. This finding is important as it suggests that although identifying the source of error (i.e. in generalizability studies) is a valid way to measure the reliability of a standard setting method [39], using the true cut-score, or an acceptable proxy of it (if real data are used), is an invaluable reference for measuring validity [57]. Consequently, this finding supports a re-thinking of the composition of Angoff panels.

The literature suggests that the Angoff judges should be experts [32], yet it recognises that experts are more stringent and may have greater influence on other judges [10, 64, 66, 70]. Fig. 3 provides some insight into this discrepancy by demonstrating the interaction between Stringency and Accuracy (being an expert). It seems that panels that are neither too stringent nor too lenient are more accurate as they are less prone to bias. However, the level of Accuracy (individual’s ability to estimate the correct cut-score) has only small impact on the panel’s cut-score *precision*. This is plausible, since the cut-score is determined by the mean of all judges’ scores [30]. Without bias in the judgement (assuming Stringency is held constant), the mean score achieved by the judges gets closer to the true value as the number of judges increases [30]. The impact of Stringency on *precision* is obvious (as it was one of the simulation parameters) but it also suggests that a panel which has only experts or only non-experts would yield a cut-score which is less *precise* than a cut-score yielded by a mixed-expertise panel (Fig. 3), particularly given the already-documented association between stringency and expertise [10]. Overall these findings suggest that optimal composition of an Angoff panel should include a diverse range of judges in terms of expertise and stringency (if known). Given the small impact of judge agreement on cut-score *precision* (variance explained = 5.1 %), this practice is recommended despite the likelihood of increasing within-panel judges disagreement.

This study found that the impact of a second Angoff round, where judges may be influenced by others (i.e. influence of ‘Leadership’), is negligible. Although this finding was negligible even when measured by standardised effect size (Cohen’s d = −0.083) it needs to be interpreted with caution particularly since the measures are all standardised and the second round was different from the first *only by the influence of judges*. This finding is supported by previous empirical studies demonstrating minor differences between two Angoff rounds [15, 61]. Other factors, such as presentation of test data, were not included in this study. It is possible that a different weighting method would have yielded a larger impact and this should be tested in future studies. The literature justifies the second round as a way to increase agreement among the judges [28, 32, 37, 43], yet as indicated above, increasing the within-judges agreement may have little impact on cut-score *precision*, which explains the observed lack of impact of a second round on the cut-score *precision*. The inevitable conclusion from these somewhat surprising results suggests that, provided there are enough judges, the original unmodified Angoff’s method [2] is robust enough and the discussion among the panellists does not significantly improve the precision of Angoff’s cut-score.

Nonetheless, the modified Angoff methods that provide additional information on the test performance itself (e.g. item and student parameters based on IRT analyses) [15, 42, 43, 63, 68] are welcomed. Such modifications are likely to increase judges’ precision without impact on Stringency, as this additional knowledge is related to test parameters only and not to level of expertise.

### Study limitations

This study has limitations, the main one being that it is a simulation study. The validity of the findings depends on the validity of the data simulation, especially the variables and the assumptions. We assumed that the judges’ *attributes* are normally distributed, rather than non-parametric. Naturally, it is possible that a particular examination and/or particular set of examinees and/or particular set of judges in real life would have different attributes from what is described in this study and thus the recommendations of this study would not be applicable for them. However, given the large number (4900) of unique panels generated for this study and the concordance with previous results generated from real data [33], it is reasonable to believe that the findings are generalizable. Moreover, as already explained, the assumptions made in the generation of the data are grounded in educational measurement and standard settings theories and findings in practice [12, 29, 32, 35, 47, 67]. Note that as expected from a simulation study, this study measures the quality of a model rather than analysing any observed data [22].

Further research is needed to identify the impact of other features of modified Angoff methods on cut-score precision, as well as repeating this study using modified assumptions.

## Conclusions and practical advice

This study demonstrates that Angoff is not only a reliable method, as previously suggested, but it is also a valid method for setting assessment standards. There are three main practice points that emerge from this study: (1) a panel of about fifteen judges provides a reliable and valid cut-score for a test consisting of 80 items or above. However, when the number of items is fewer than 15, it is recommended that no fewer than 20 judges are used, with the number of judges increasing as the number of items declines; (2) panels should include judges with mixed levels of expertise unless there is clear evidence that the experts among the panellists are not more stringent than the non-experts; and (3) without providing additional test data to the judges, a second round of Angoff is redundant.

Lack of data of the true ability of examinees remains a major hurdle in the field of standard setting. Thus research utilising simulation methods may enhance our understanding about the validity and applicability of a range of standard setting methods beyond what was demonstrated in this study.

## Ethics approval and consent to participate

This study used computer generated data i.e. simulated data, hence consent to participate was not necessary. Thus, ethics approval was not required.

## Availability of supporting data

Data used in this study may be available by request. Please contact A/Prof Boaz Shulruf b.shulruf@unsw.edu.au.
